# Tuberculosis in older adults: case studies from four countries with rapidly ageing populations in the western pacific region

**DOI:** 10.1186/s12889-023-15197-7

**Published:** 2023-02-21

**Authors:** Alvin Kuo Jing Teo, Kalpeshsinh Rahevar, Fukushi Morishita, Alicia Ang, Takashi Yoshiyama, Akihiro Ohkado, Lisa Kawatsu, Norio Yamada, Kazuhiro Uchimura, Youngeun Choi, Zi Chen, Siyan Yi, Manami Yanagawa, Kyung Hyun Oh, Kerri Viney, Ben Marais, Heejin Kim, Seiya Kato, Yuhong Liu, Catherine W.M. Ong, Tauhid Islam

**Affiliations:** 1 Saw Swee Hock School of Public Health, National University of Singapore and National University Health System, Singapore, Singapore; 2 Faculty of Medicine and Health, University of Sydney, Sydney, NSW Australia; 3 World Health Organization, Regional Office for the Western Pacific, Manila, Philippines; 4 Division of Infectious Diseases, Department of Medicine, National University Hospital, Singapore, Singapore; 5 Research Institute of Tuberculosis, Anti-Tuberculosis Association, Tokyo, Japan; 6 Korean National Tuberculosis Association, Seoul, Republic of Korea; 7 Office of International Cooperation, Innovation Alliance on Tuberculosis Diagnosis and Treatment, Beijing, China; 8 KHANA Center for Population Health Research, Phnom Penh, Cambodia; 9 Center for Global Health Research, Public Health Program, Touro University California, Vallejo, CA USA; 10 Global Tuberculosis Programme, World Health Organization, Geneva, Switzerland; 11 The University of Sydney Institute for Infectious Diseases (Sydney ID) and the Centre of Research Excellence in Tuberculosis (TB-CRE), Sydney, NSW Australia; 12 Beijing Chest Hospital, Capital Medical University, Beijing, China; 13 Infectious Diseases Translational Research Programme, Department of Medicine, National University of Singapore, Singapore, Singapore; 14 Institute of Health Innovation and Technology (iHealthtech), National University of Singapore, Singapore, Singapore; 15 Division of Infectious Diseases, Department of Medicine, Woodlands Health, Singapore, Singapore

**Keywords:** Aging, Best practices, Challenges, Policy, China, Japan, Republic of Korea, Singapore

## Abstract

**Background:**

The Western Pacific Region has one of the fastest-growing populations of older adults (≥ 65 years) globally, among whom tuberculosis (TB) poses a particular concern. This study reports country case studies from China, Japan, the Republic of Korea, and Singapore reflecting on their experiences in managing TB among older adults.

**Findings:**

Across all four countries, TB case notification and incidence rates were highest among older adults, but clinical and public health guidance focused on this population was limited. Individual country reports illustrated a range of practices and challenges. Passive case finding remains the norm, with limited active case finding (ACF) programs implemented in China, Japan, and the Republic of Korea. Different approaches have been trialled to assist older adults in securing an early diagnosis, as well as adhering to their TB treatment. All countries emphasised the need for person-centred approaches that include the creative application of new technology and tailored incentive programs, as well as reconceptualisation of how we provide treatment support. The use of traditional medicines was found to be culturally entrenched among older adults, with a need for careful consideration of their complementary use. TB infection testing and the provision of TB preventive treatment (TPT) were underutilised with highly variable practice.

**Conclusion:**

Older adults require specific consideration in TB response policies, given the burgeoning aging population and their high TB risk. Policymakers, TB programs and funders must invest in and develop locally contextualised practice guidelines to inform evidence-based TB prevention and care practices for older adults.

**Supplementary Information:**

The online version contains supplementary material available at 10.1186/s12889-023-15197-7.

## Background

Globally the number of older adults (aged ≥ 65 years) is expected to triple by 2100 [1]. Life expectancy at birth has gradually increased since the 1950s, [1] with the number of older adults projected to exceed children aged < 5, across all economies by 2020 [2]. The Western Pacific Region has one of the fastest-growing populations of older adults globally. In 2019, life expectancy at birth of the population was 4 years above the global estimate of 73.3 years [3]. Japan, for instance, is the most aged country in the world (average life expectancy 85 years) [4]. Several other countries in the region, particularly the People’s Republic of China (hereinafter referred to as China), the Republic of Korea, and the Republic of Singapore (hereinafter referred to as Singapore), were estimated to have an increase in the proportion of older adults globally between 2019 and 2050.

Japan has the highest proportion of older adults in the world, at 28% of the total population in 2020, [5] and the proportion is expected to rise to 38% by 2065 [6]. China is also undergoing a profound demographic transition. In 2021, the number of older adults reached 191 million, amounting to 13.5% of the total population. The number rose by 4.6% compared to the previous year, [7] and the increasing trend is likely to persist with the number of older adults expected to exceed 480 million (34.6% of the total population) by 2050 [8]. Similarly, the demographic transition in the Republic of Korea has seen rapid growth in the older adult population. In 2020, 16% of the total population was ≥ 65 years; this is expected to reach 37% by 2050 [9]. The older adult population made up 13% of the total population in Singapore in 2020 [5]. The proportion is projected to rise on the same trajectory as China, Japan, and the Republic of Korea in the next few decades [1].

**Fig. 1:**
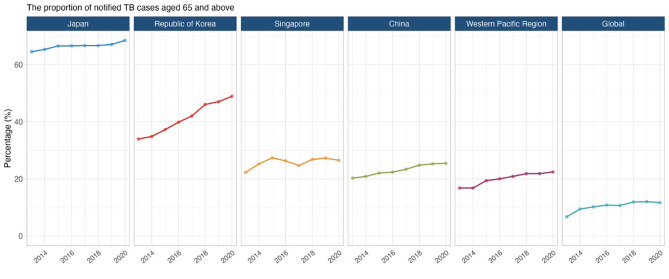
The proportion of notified TB cases aged ≥ 65 in Japan, the Republic of Korea, Singapore, China, the Western Pacific region, and globally 2013−2020 Data source: World Health Organization Global TB Programme 2021

Improvements in life expectancy have been attributed to better nutrition, political stability, risk factor reduction and improved healthcare access [10]. Globally, older adults contributed 26% of the total disease burden (measured in disability-adjusted life years [DALYs]); 38% of the burden in high and upper-middle-income, and 16% in low-and lower-middle-income regions in 2019 [11]. Non-communicable diseases such as cancer, cardiovascular disease and neurological disorders accounted for most of this burden, but respiratory infections, including tuberculosis (TB), are a major contributor as well [11]. In fact, TB case notifications and estimated disease incidence rates were highest among older adults in China, Japan, the Republic of Korea, and Singapore (Fig. 1) [12].

With increasing age, progressive immune dysfunction (immunosenescence) increases the risk for TB disease development [13, 14]. The convergence of co-morbidities such as diabetes, chronic respiratory disease and undernutrition, and lifestyle behaviour like tobacco smoking also increase TB risk [15–18]. Considering the burgeoning aging population in the Western Pacific Region and the limited guidance specific to this population, it is clear that the needs of older adults require greater recognition in TB response policies. This study reports country case studies from China, Japan, the Republic of Korea, and Singapore, reflecting on country-specific experiences in TB diagnosis and management among older adults. The case studies are a part of a broader endeavour, including a narrative review and analysis of epidemiological trends, to understand and document TB management among older adults in the region. The findings from the narrative review (see supplementary materials for methods) were used to support the country-specific experiences reported in the case studies.

### China

#### TB epidemiology

China has the second-highest TB burden globally. In 2020, China had an estimated 842,000 people with TB (59 per 100,000 population per year), with older adults accounting for a quarter of all cases [12]. China notified 159,100 cases (19% of all notified cases) among older adults, [12] and a consistently increasing trend has been observed over the last two decades [19]. The fifth national TB epidemiological survey performed in 2010 recorded prevalence rates for clinical and bacteriologically confirmed TB as 482 and 138/100,000 population, respectively, among older adults [20]. The TB prevalence rate in the survey increased with age and peaked in the 75−79 year age group at 866/100,000 population (overall prevalence rate: 442/100,000 population [95% CI 417−469]) [20]. A subsequent prospective cohort study reported high TB incidence rates, [21] highlighting the need to prioritise older adults in the TB response. TB-related mortality was also more prevalent among older adults [19] concentrated in China’s Central and South-Eastern regions, [22] among lower-income residents from rural areas and in those with comorbidities [23].

#### Health system structure and TB services

The Government of China introduced directly observed treatment (DOT) (now known as treatment support), [24] as a strategy, in 13 provinces in 1991, with a nationwide scale-up by 2001 [25, 26]. The National Center for Disease Control (CDC) is responsible for coordination, policymaking, standards and technical guidance, while provincial CDCs conduct TB programs and monitor disease trends at the prefecture and county level.

TB diagnosis and treatment are provided under the free-TB service policy in China [27, 28]. The policy is implemented through an integrated model where designated hospitals (specialised centres for infectious diseases and general hospitals with TB departments) provide TB diagnosis and treatment services [29]. Persons with drug-resistant TB are treated at designated prefectural or provincial-level hospitals [29]. Primary healthcare providers are responsible for referring older adults presenting with TB symptoms to designated county-level hospitals for diagnostic investigation. They also conduct follow-up, case management and support, and health education activities at the community level [29]. The private sector is not involved in TB management. This free-TB service policy is supported by funds from the central and local governments [29]. Expenditures incurred beyond the free-TB service policy are generally covered by other government-linked insurance schemes [27]. However, the insurance and financial support schemes vary between provinces, resulting in different levels of financial reimbursement.

Despite this policy, catastrophic costs due to TB remain substantial. Studies report that more than 50% of TB patients incurred out-of-pocket payments exceeding 40% of the household’s ‘capacity to pay, [30, 31] indicating a need for better financial support plans and packages to be considered. In addition to financial support, social support through better health education, supportive services, and community engagement is important to improve TB treatment adherence and outcomes [32].

#### TB case finding

Active case finding (ACF), a provider-initiated systematic screening and testing for TB disease, [33] has been deployed in conjunction with the annual physical assessments program for older adults since 2015. Such an integrated approach was feasible and effective for TB case detection among older adults, especially those with comorbidities such as diabetes [34–36]. TB management is carried out by community health service centres under the technical guidance of the National Tuberculosis Plan [37, 38].

Institutionalised aged care is a booming sector in China. As of September 2021, more than 2.6 million older adults were living in retirement facilities [39]. The quality of services provided in these facilities provides is inconsistent, often resulting in over-crowding in poorly ventilated conditions. Consequently, disease outbreaks are common and in one TB outbreak, 40% of the residents were diagnosed with TB disease [40]. Therefore, amendments to existing legislation regarding infection prevention and control in such facilities should be considered, with relevant TB information provided to residents and staff. ACF should be prioritised with the expansion of the annual physical assessments program.

#### TB care

TB treatment, care and support have been decentralised using various enablers such as the community, family members, local primary healthcare providers, and technology (electronic medication monitor) for people with TB who were clinically stable and non-infectious [26, 41]. However, given their advancing age, existing chronic health conditions, and weakened immunity, older adults are prone to stopping treatment (loss to follow-up) and death [42]. Currently, limited guidance is provided at the country and global levels to address issues encountered by older adults, such as adverse event monitoring and management, drug-drug interactions and treatment adherence.

Traditional Chinese herbal medicines have been used to treat TB and as adjuvants to improve the tolerability of TB treatment. Although not endorsed by the WHO, herbal medicines are widely used in China [43]. In 2020, a meta-analysis reported some potential benefits, [44] but unfortunately, the evaluated studies lacked methodological rigour. Given how widely herbal medicines are used in China, especially in older adults, practice guidelines should consider how best to advise and regulate health practitioners.

#### TB prevention

The ramp-up of TB response through nationwide expansion of treatment support (historically known as directly observed treatment [DOT]) and improvement of disease reporting and referral system saw a 28% and 65% reduction in the prevalence of pulmonary TB and smear-positive TB, respectively, between 1990 and 2010 in China [45]. As the risk of infection and transmission decreases, it is expected that reactivation will be the main driver of TB disease occurrence as the population ages in the coming decades [46, 47]. China has around 350 million people with TB infection, [48] however, screening for TB infection and the uptake of TB preventive treatment (TPT) among at-risk groups is limited. Incorporation of TB infection screening into the annual health assessment could improve awareness regarding TB infection and uptake of TPT among at-risk groups but requires further evaluation for feasibility and safety [49].

### Japan

#### TB epidemiology

The estimated TB incidence in Japan has declined from 36 to 12/100,000 population per year from 2000 to 2020 [12]. The treatment coverage (notification rate as a proportion of the estimated incidence) was approximately 85%, with 69% reported among older adults (43% among those ≥ 80 years) [12, 50]. The median age of a person notified with TB in 2020 was 77 years [50]. While the number and rate of TB cases have decreased in the last decade, the proportion of older adults among new cases has increased from 48% to 2000 to 59% in 2010 and 69% in 2020 [50]. Although this is mainly driven by reactivation, multiple outbreaks with transmission in health and aged-care facilities have been reported [51, 52]. Despite increased rates of disease, TB mortality among older adults has declined from > 10 to < 5 deaths per 100,000 population in recent years [53]. Nevertheless, the highest number of TB deaths occurred among older adults in Japan [53].

#### Health system structure and TB services

The Ministry of Health, Labour, and Welfare of Japan comprises 18 departments, bureaus, and offices that oversee public health, workplace safety and sanitation, medical services, and health insurance [54]. Access to healthcare in Japan is facilitated by a universal health insurance scheme with population-wide coverage. While approximately 80% of the health facilities are privately owned, they are regulated by the government, and payment for services is controlled by the ministry under the insurance scheme [54]. These facilities provide direct medical care for people with TB, including in-patient care during TB treatment. Meanwhile, the public health centres provide other aspects of TB response such as screening for TB disease and infection, contact investigation, surveillance, and community-based treatment support [55]. National TB surveillance data are managed by the Research Institute of Tuberculosis [55].

TB care is covered by national health insurance and public subsidies at about 70% and 25%, respectively. The remaining 5% is paid out-of-pocket, but a public assistance fund can be used to offset costs for those who cannot afford it [55]. Medical expenses incurred during hospitalisation are wholly covered by health insurance and public subsidies. Given the rising need for dedicated and specialised care for older adults, a long-term care insurance scheme was introduced in 2020 to support those in need, as well as their family members [54].

#### TB case finding

In Japan, TB among older adults has historically been detected passively at outpatient and inpatient health facilities [56, 57]. TB case finding among older adults in the health facilities were facilitated by the presence of comorbidities and routine follow-up for other health conditions, thus increasing the likelihood of care-seeking at the onset of TB symptoms and, subsequently, screening for TB [58]. However, case finding in such settings hinge on a clinician’s awareness of the need to screen for TB. On presentation to health facilities, TB diagnosis among older adults has historically been difficult due to atypical presentation in older adults. For example, one study reported that > 40% of older adults with TB presented with atypical features of TB in Japan [59]. The non-specific symptoms and low awareness of TB [60], especially in intermediate TB burden settings such as Japan, could lead to delayed diagnosis.

In addition, community-based ACF programs targeting older adults have been recommended and implemented [61]. Currently, these programs prioritise older adults ≥ 80 years for TB screening using mobile chest x-rays in the community. For institutionalised older adults, those aged ≥ 65 are offered annual TB disease screening to prevent outbreaks in aged-care facilities. Before admission to an aged-care facility, TB screening using chest radiography has also been implemented to facilitate the early detection of TB disease [62]. This obligation is also extended to frontline workers in health and aged-care facilities and welfare facilities.

#### TB care

Treatment of drug-susceptible TB in Japan generally follows the standard 6-month regimen [55] . However, a 9-month regimen without pyrazinamide (2 months of isoniazid, rifampicin, and ethambutol, followed by isoniazid and rifampicin for 7 months) is widely prescribed for older adults, particularly those aged ≥ 80, and the national guidelines did not recommend pyrazinamide for older adults with TB until 2018 [63]. While recent studies have reported that the pyrazinamide-based regimens do not lead to significantly higher rates of treatment discontinuation, liver injuries, and death than regimens without pyrazinamide, [63, 64] the proportion of those receiving the standard 6-month regimen with pyrazinamide declines with age, particularly for those aged ≥ 75 years [50].

The overall treatment success rate of drug-susceptible TB was 66% in Japan in 2019 [50] . The treatment success rate among people with TB < 65 years was 82%, and the rate decreased among the older age groups (65−74 years; 76%, 75−84 years; 65%, ≥85 years; 46%) [50]. The low treatment success rate among older adults has been ascribed to a high death rate during TB treatment. In 2019, 33% of older adults with TB died during treatment, and > 60% died of non-TB-related causes [50]. Japan has a comprehensive treatment support strategy for people with TB disease or TB infection (TPT). For those who require in-patient care, treatment support that comprises patient education is provided in the hospital. Upon discharge, an individual support plan for community-based treatment support is developed based on the person’s risk of non-adherence, and the frequency and means of medication support are determined. A treatment support conference is held to evaluate and review the plan. Through a patient-centred care approach, treatment support may be provided by health professionals in the local community, community health workers and volunteers, or family members.

#### TB prevention

Tuberculin skin tests (TST) and interferon-gamma release assays (IGRA) are used to diagnose TB infection in Japan; both tests are covered by national health insurance. TB infection has been a notifiable condition since 2006 [50]. While there are no policies to actively screen older adults as a priority group for TB infection, notification of TB infection among older adults ≥ 65 has gradually increased since 2010 [50]. In 2020, approximately 49% of older adults with TB infection were detected via contact investigation, [50] a national policy to facilitate early detection of TB disease and infection and prevent onward transmission. There was also an increasing number of older adults diagnosed with TB infection in the hospital due to TB infection testing carried out before treating other medical conditions such as rheumatoid arthritis [65]. Nevertheless, the lower sensitivity of IGRA among older adults remains a concern. A 2017 study in Japan reported discrepancies between IGRA positivity rates and the corresponding estimated prevalence of TB infection among older adults, highlighting the utility and applicability of IGRA in this population [66]. Furthermore, providing TPT for older adults remains controversial among clinicians due to the risk of adverse events.

### Republic of Korea

#### TB epidemiology

In 2020, in the Republic of Korea, there was an estimated TB incidence was 25,000 people with incident TB (49 per 100,000 population per year); 94% of these were notified to the national authorities, of which 49% were aged ≥ 65 [12]. [12] While the number of people with TB has steadily declined in the last decade, the proportion of older adults among new TB patients has risen (19.2% in 2001 and 49.1% in 2020) [67] . In 2020, the TB notification rate among older adults aged 65−69 was 58 per 100,000 population; the rate increased with age and peaked among those aged ≥ 80 years at 235 per 100,000 population [67]. TB mortality among older adults ≥ 65 was 13.8 per 100,000 population in 2020, the lowest rate recorded since 2001. However, of all TB-related deaths, 82.5% involved those aged ≥ 65, and the proportion has been above 80% since 2016 (2001; 58.0%, 2010; 72.1%). Despite the decline in overall TB burden and mortality rates, the course is predicted to reverse after 2032 (TB incidence) and 2026 (TB deaths) due to increasing trends among older adults, particularly those aged ≥ 80 69.

#### Health system structure and TB services

The Ministry of Health and Welfare is the national policymaking and governing body for public health and medical services in the Republic of Korea [70]. The ministry also oversees the national health insurance scheme, a compulsory scheme that confers health care coverage and benefits for the entire population [70]. The Korean Disease Control and Prevention Agency (KDCA) is responsible for disease surveillance, public health response, disease prevention, and research and oversees the National TB Elimination Project [70]. While policies regarding TB prevention and care are established by the government, the private sector’s involvement in TB care began in the 1990s and was formalised in 2011 through the public-private mix model [71].

TB care in the public and private sectors is covered by national health insurance. A 10% co-payment scheme was in place until 2016 [71]. Since 2017, all expenses incurred during TB care, including hospitalisation, isolation orders (movement restriction to prevent further transmission), and an allowance for dependents, are included in the policy, thereby minimising out-of-pocket payments for people affected by TB. Interventions such as contact investigation and the screening of close contacts for TB disease and infection are also covered by health insurance. While there is specific funding for TB interventions targeted at older adults in the Republic of Korea, the budget only amounted to 1.8% of the total budget for the national TB control program in 2018 [71].

#### TB case finding

Since the inception of the national TB control program in the 1960s, early detection and treatment of TB has been a mainstay of TB policies, including for older adults [71]. In 2020, the TB incidence rate detected through TB screening among older adults ≥ 65 using mobile chest x-ray machines in 17 regions and provinces was 75 per 100,000, 1.9 times higher than the general population’s rate [72]. This high detection rate prompted KDCA to implement nationwide TB screening among older adults in the community and aged-care facilities as a key strategy to detect TB early in this group.

With an increasingly aging population and the growth in the proportion of women in the labour market, the number of older adults living in institutionalised settings has also increased proportionally, partly due to the introduction of long-term care insurance, which covers home and institutional care services, assistive equipment such as walker and wheelchair, and cash benefits [73]. In fact, the number of recipients of long-term care insurance increased three-fold between 2008 and 2019 [74]. Using mobile chest x-ray, TB screening among older adults ≥ 65 in long-term care facilities in 17 regions and provinces resulted in an incidence rate of 66 per 100,000 in 2020 [72].

#### TB care

Drug-susceptible TB in the Republic of Korea is treated using the standard 6-month regimen [75]. Partly due to concerns about potential pyrazinamide hepatotoxicity, there is ongoing work to optimise TB treatment among older adults through establishing an adverse events monitoring system, managing adverse events, and developing biomarkers that may predict diagnostic and therapeutic responses [76].

Overall, the TB treatment success rate has been sitting at approximately > 80% over the last two decades [12]. However, the treatment success rate among older adults was lower than the general population at approximately 70% between 2012 and 2015 [77]. Treatment support remains the main treatment administration option. Video-observed therapy and support through a comprehensive support centre for older adults living alone had been trialled with some success. Preliminary data showed higher treatment success rate among older adults enrolled in the programme [78]. Other incentives have been implemented, such as providing food, daily necessities, and medical accompaniment service for older adults who have trouble remaining on treatment. Furthermore, an approach to assess the vulnerability of people with TB, particularly older adults, with customised case management and linkage with social welfare services have also been implemented.

#### TB prevention

A comprehensive epidemiologic and contact investigations protocol is in place to screen at-risk populations for both TB disease and infection. TB disease and infection screening is mandatory for healthcare and nursery workers and teachers. Other at-risk groups, such as people living with HIV, those with silicosis, organ transplant recipients, and patients with kidney disease on dialysis, are recommended to be screened for TB infection. For household contacts of persons with TB, chest x-ray investigations and TB infection workups (TST/IGRA, but not mandatory) will be offered (covered by health insurance). TPT (3HP [isoniazid-rifapentine once-weekly for 3 months], 3RH [rifampicin-isoniazid daily for 3 months], or 4R [rifampicin daily for 4 months) is offered to those eligible for it. However, there is no policy to screen older adults for TB infection. In a 2016 study conducted across 11 regions, 40% of those aged ≥60 tested positive for TB infection using IGRA [79]. Considering the potentially high prevalence of TB infection and risk for reactivation among older adults, [80] a systematic approach to TB infection screening and TPT administration in this population is warranted.

### Singapore

#### TB epidemiology

An estimated 2700 people were affected by TB in 2020 (46 per 100,000 population per year); 89% of these (2400) were notified to the national authorities in 2020. [12] Of the 2400 people with TB, approximately 57% were Singapore citizens and permanent residents [81]. The TB incidence rate has been approximately 40 per 100,000 since the late 1990s and may be attributed to migration (about 30−50% of the cases were detected among short and long-term visitors, including work pass holders and students) and an aging population [82, 83]. In 2020, older age groups (≥60) made up a significant proportion of the TB notifications both among Singaporean-born (58%) and foreign-born (46%) individuals [81]. While reactivation of past infections could sustain the TB epidemic among older residents, outbreaks in aged-care facilities have also been reported. Overall, TB mortality rates have remained < 1 per 100,000 population in the past few years [12]. However, the TB mortality rate among older adults ≥70 was higher at 5.1 per 100,000 population in 2018 [84].

#### Health system structure and TB services

The Ministry of Health (MOH) Singapore initiated the national TB program, named Singapore TB Elimination Program (STEP), to strengthen TB response efforts by detecting and treating TB disease and infection and preventing drug-resistance TB in 1997 [82]. STEP manages a notification registry and a treatment surveillance system to monitor TB notification and treatment outcomes. The TB Control Unit (TBCU) is the referral centre for TB management in Singapore, responsible for treatment support, contact investigation, and the administration of TPT. Both public and private hospitals can offer TB diagnosis and treatment. Referrals for TB diagnosis and treatment can be made by primary care physicians in public, private, and community-based organisations (such as the Singapore Anti-Tuberculosis Association).

Singapore’s health system is financed through subsidies, a national health savings account (MediSave), a basic national health insurance scheme (MediShield) for hospitalisation and treatment bills, and an endowment scheme for individuals who have exhausted other means of payment [85]. For citizens and permanent residents, TB diagnosis and treatment costs are covered by the health financing system. Non-residents, such as those holding long-term work visas, are not covered by these schemes and must rely on employer coverage, private health insurance, or out-of-pocket payment for TB screening and diagnosis costs. TB medications are provided to all at no cost.

#### TB case finding

TB is diagnosed by passive case finding when a person presents with symptoms or a chest x-ray for other medical conditions (yet suggestive of TB) [86]. TB screening is conducted for foreigners applying for work or long-term visas and during the visa renewal process using chest radiography [86]. The same approach applies to everyone regardless of age. There are currently no specific interventions targeting TB screening among older adults. Some aged-care facilities may enquire about the date and information of the last chest x-ray or require a chest x-ray to be conducted before admission. However, this policy is inconsistent.

#### TB care

Treatment of drug-susceptible TB in Singapore follows the standard 6-month regimen (2 months of isoniazid, rifampicin, ethambutol, and pyrazinamide followed by isoniazid and rifampicin for 4 months) [86]. However, for older adults who may be unlikely to tolerate pyrazinamide, a 9-month regimen comprising ethambutol, rifampicin, and isoniazid for 2 months (intensive phase), followed by rifampicin and isoniazid for 7 months (continuation phase) is used [86]. It is required by law under the Infectious Diseases Act for all clinicians to report treatment progress and the outcomes of people with TB to the MOH [86].

The treatment success rate was 79% in 2019, [12] and the treatment success rate among older adults was not known at the time of writing. Different forms of treatment support have been implemented to support treatment. Outpatient treatment support is implemented at all the 18 public polyclinics and the TBCU. People with TB disease can choose to have their treatment administered at the nearest public health facility. In a 2016 study among adults with TB in Singapore, 72% of respondents reported that they could accept the schedule of facility-based treatment support [87]. However, 45% perceived the arrangement to be disruptive to their work, school, and social activities [87]. The results were not age disaggregated. While not specific to older adults, incentive-based intervention to encourage treatment adherence among people with TB from the lower-income bracket has shown promise compared to the non-intervention group in Singapore [88]. Other forms of treatment support, such as administration in aged-care facilities and through outreach for recipients who have mobility issues or are frail, have been implemented [86]. Self-administered treatment is also used in Singapore, especially for those treated in the private sector. However, there are no data on self-administered treatment among older adults.

#### TB prevention

The STEP strategy includes contact investigation to identify close contacts of persons with bacteriologically confirmed TB [86]. Testing for TB infection is recommended for high-risk groups if there is an intention to treat with TPT [86]. IGRA is the preferred test for TB infection; variable TPT options are available, including 6 H (isoniazid daily for 6 months), or 4R (daily rifampicin for 4 months) [86]. Although TB infection has been estimated to affect up to 30% of older adults in Singapore, [81] there is no policy or specific guideline regarding TPT use for older adults. The risk of adverse events associated with TPT remains a concern, and further studies are needed.

## Summary and conclusion

The four case studies presented exemplify the range of practices and challenges in managing TB among older adults in the Western Pacific Region. Here we summarise the key findings and action points based on country experiences:

1. While passive case finding remains the mainstay intervention contributing to TB case detection in older adults, routine ACF has been implemented in China, Japan, and the Republic of Korea. The optimal use of ACF requires further consideration and assessment in different contexts.
2. A person-centred approach to TB care was identified as a key theme in all four countries. Nevertheless, limited guidance is available at the country and global levels to address issues older adults encounter (e.g., adverse event monitoring and management, consideration of drug-drug interactions, and treatment adherence). Existing evidence (and evidence gaps) related to the optimal management of older adults with TB require systematic review and guideline development.
3. There is a lack of standardisation in the approach to TB infection testing and TPT provision. It is important to generate the necessary evidence to inform benefit-risk estimates for managing TB infection among older adults.
4. The use of traditional medicines is culturally deeply rooted among older adults in this region. Their complementary use in TB care should be explored and carefully considered.

**Fig. 2:**
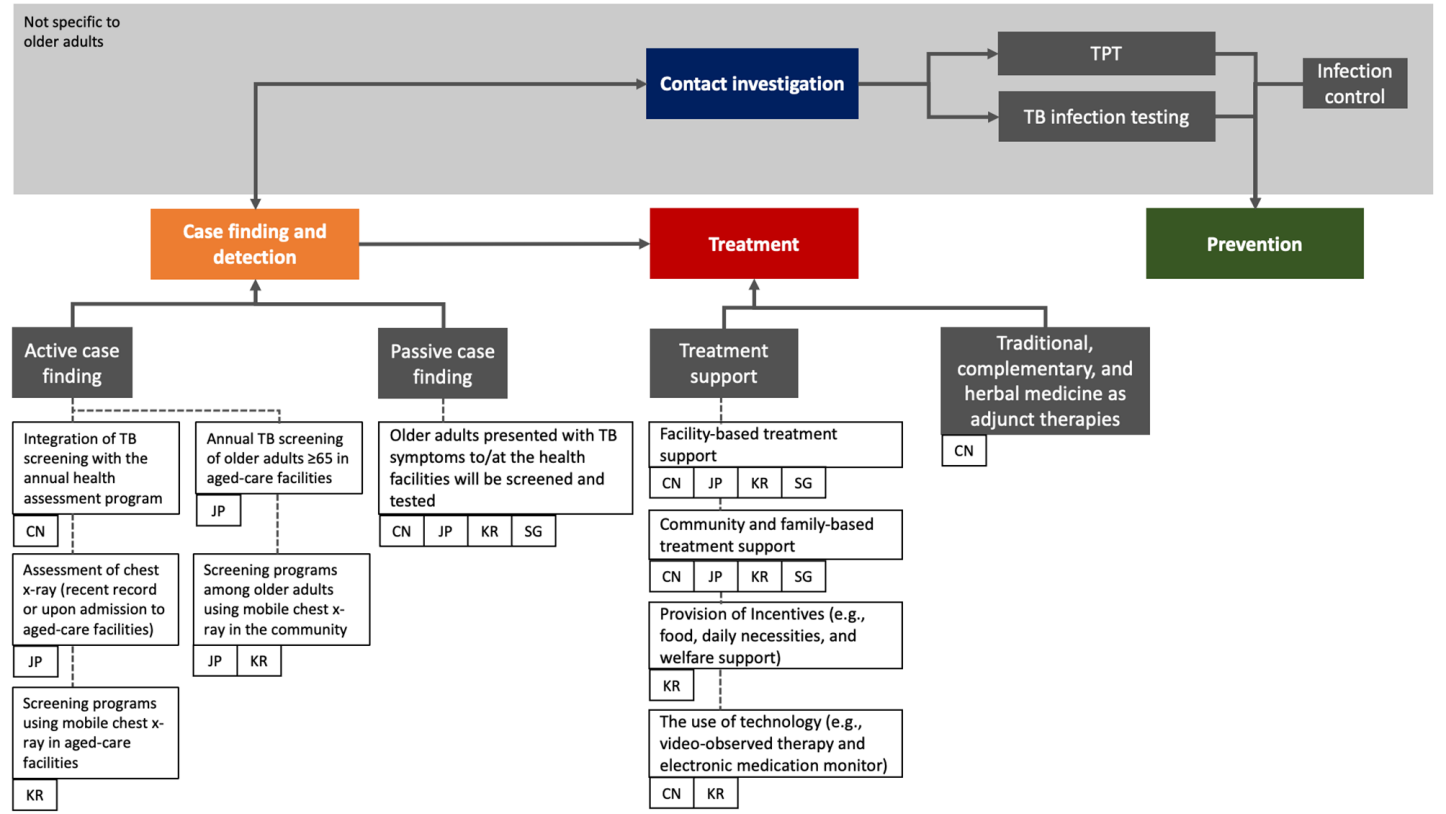
TB management among older adults in China, Japan, the Republic of Korea, and Singapore TB management experiences were mapped based on three main domains: case finding and detection, treatment, and prevention. The relevant policies and interventions are tagged to the respective implementing countries. Areas shaded in grey represent cross-cutting interventions that extend beyond the older adult population. Treatment support was historically known as directly observed treatment (DOT) Abbreviations: TB; tuberculosis, TPT; TB preventive treatment, CN; China, JP; Japan, KR; Republic of Korea, SG; Singapore

A summary of the approaches implemented by the four countries is outlined in Fig. 2.

### Cross-cutting challenges

The COVID-19 pandemic has severely disrupted access to TB care. with an 18% decrease in TB disease notifications globally [89]. Health services for older people were often more severely.

affected and TB services in South-East Asia and the Western Pacific Regions were highly impacted [12]. Of the four countries included in this study, TB notification data for 2020 and 2021 were lower than 2019 [12, 90].

In addition to key risk factors for TB such as living in crowded and poorly ventilated conditions, indoor air pollution and tobacco smoking, [91] older adults also have age-associated immune dysfunction, financial resources constraints, and frequent comorbidities [92]. Caring for older adults with TB can be difficult and complex cases may require referral to appropriate specialty care. Service integration between relevant specialties should facilitate better care for older adults with TB. TB diagnosis in older adults is further complicated by challenges related to cognitive impairment, communication challenges and difficulties in obtaining quality sputum samples [93, 94]. Dementia, which primarily affects older adults, has been associated with difficult TB diagnosis and poor adherence to TB treatment in the absence of tailored treatment support [95].

TB-related stigma (perceived and experienced) remains pervasive in many communities [96]. While systematic assessment of TB-related stigma among older adults has not been conducted, older adults in China have reported unwillingness to seek TB care due to fear of discrimination and self-isolation because of stigma [32, 97]. Anecdotally, aged care facilities in Japan were reluctant to accept returning residents after hospital admission for TB care due to fear and stigma, while unwillingness to be screened for TB has also been ascribed to stigma.

The high standards of living and well-being may have shaped the notion that TB is not a significant concern, rendering a lower index of TB suspicion, including among healthcare workers [98]. A recent cross-sectional study conducted among older adults in Shenzhen, China, reported that > 70% of the respondents were aware of TB as a contagious disease, its symptoms, and preventive measures. Yet, less than half knew that TB was curable, and only one-third were willing to be screened for TB if they were to develop suspicious symptoms [99].

### Conclusion

Considering that aging populations is a global phenomenon that implies a heightened TB risk, particularly in areas with historically high rates of TB, policymakers and funders must invest more to deliver and generate the evidence required to inform optimal TB prevention and care initiatives in older adults.

## Electronic supplementary material

Below is the link to the electronic supplementary material.

Supplementary Material 1

## Data Availability

Data used for this case study comprised published literature, reports, and co-authors’ experience in managing TB among older adults in their respective settings.
