# Correction: Tuberculosis in older adults: case studies from four countries with rapidly ageing populations in the western pacific region

**DOI:** 10.1186/s12889-023-15576-0

**Published:** 2023-06-07

**Authors:** Alvin Kuo Jing Teo, Kalpeshsinh Rahevar, Fukushi Morishita, Alicia Ang, Takashi Yoshiyama, Akihiro Ohkado, Lisa Kawatsu, Norio Yamada, Kazuhiro Uchimura, Youngeun Choi, Zi Chen, Siyan Yi, Manami Yanagawa, Kyung Hyun Oh, Kerri Viney, Ben Marais, Heejin Kim, Seiya Kato, Yuhong Liu, Catherine W. M. Ong, Tauhid Islam

**Affiliations:** 1 Saw Swee Hock School of Public Health, National University of Singapore and National University Health System, Singapore, Singapore; 2 Faculty of Medicine and Health, University of Sydney, Sydney, NSW Australia; 3 World Health Organization, Regional Office for the Western Pacific, Manila, Philippines; 4 Division of Infectious Diseases, Department of Medicine, National University Hospital, Singapore, Singapore; 5 Research Institute of Tuberculosis, Anti-Tuberculosis Association, Tokyo, Japan; 6 Korean National Tuberculosis Association, Seoul, Republic of Korea; 7 Office of International Cooperation, Innovation Alliance on Tuberculosis Diagnosis and Treatment, Beijing, China; 8 KHANA Center for Population Health Research, Phnom Penh, Cambodia; 9 Center for Global Health Research, Public Health Program, Touro University California, Vallejo, CA USA; 10 Global Tuberculosis Programme, World Health Organization, Geneva, Switzerland; 11 The University of Sydney Institute for Infectious Diseases (Sydney ID) and the Centre of Research Excellence in Tuberculosis (TB-CRE), Sydney, NSW Australia; 12 Beijing Chest Hospital, Capital Medical University, Beijing, China; 13 Infectious Diseases Translational Research Programme, Department of Medicine, National University of Singapore, Singapore, Singapore; 14 Institute of Health Innovation and Technology (iHealthtech), National University of Singapore, Singapore, Singapore; 15 Division of Infectious Diseases, Department of Medicine, Woodlands Health, Singapore, Singapore

**Correction: BMC Public Health 23, 370 (2023)**

**https://doi.org/10.1186/s12889-023-15197-7**

Following publication of the original article [1], the authors identified 2 errors:

1. In the background the sentence “In 2019, life expectancy at birth of the population was 4 years above the global estimate of **73.7 years**” should read In 2019, life expectancy at birth of the population was 4 years above the global estimate of **73.3 years**
2. The following disclaimer was missing: **The authors alone are responsible for the views expressed in this article, and they do not necessarily represent the views, decisions, or policies of the institutions with which they are affiliated.**

The original article has been updated.
