# Supplementary material for: Tuberculosis in older adults: case studies from four countries with rapidly ageing populations in the western pacific region
Source: BMC Public Health. 2023 Feb 21;23:370. doi: 10.1186/s12889-023-15197-7 (PMC9942033; doi:10.1186/s12889-023-15197-7)
Supplement: Supplementary file 1 — Supplementary Material 1 [file 12889_2023_15197_MOESM1_ESM.docx]

# **Supplementary materials**

# **Tuberculosis in older adults: case studies from four countries with rapidly ageing populations in the Western Pacific Region**

We used findings from a narrative review performed separately to support the experiences shared by key stakeholders (co-authors) in the 4 countries. The narrative review aimed to describe the risk factors for TB disease and transmission, as well as the clinical manifestations of TB in older adults. The review also aimed to highlight key challenges and associated strategies, as well as research priorities, in TB transmission, detection, diagnosis, treatment and post-TB health among older adults in the Western Pacific Region.

This study focused on case studies instead of the narrative review. Therefore, we present methods used for the narrative review methods as supplementary information as follows:

# **Methods**

We conducted a narrative review of the available literature on PubMed published between 1996 and 2021. There were no language restrictions, and search phrases comprised a combination of “aging”, “ageing”, “older people”, “elderly”, and “tuberculosis” (free-text index terms and Medical Subject Headings terms). Boolean operators “OR” and “AND” were used to search for articles relevant to TB in older adults.

We focused our search on country experiences reported from the Western Pacific Region, as defined by WHO.^1^ In the review of general subject topics, we included studies that described the risk factors, transmission and reactivation, and clinical manifestations of TB disease among older adults. However, due to the breadth of the topic areas covered, undertook a purposive approach in article selection of relevant articles from the region, as well as articles providing a more general overview (e.g., systematic reviews, meta-analyses, and narrative reviews). In addition, we reviewed the reference lists of selected articles and consulted a range of regional stakeholders to ensure that key areas within each domain were presented and discussed.

For country-specific experiences, we included studies that described challenges, interventions, and policies in managing TB among older adults along the care cascade in the region. Works outside the region and among populations not identified as older adults were excluded. In total, we identified 759 unduplicated records, of which 91 full texts were reviewed after initial title screening (**Supplementary** **Figure 1**). The stakeholders and co-authors reviewed multiple iterations of the narrative and reference lists. Additional references were suggested and added to ensure completeness and relevance to the specified scope of the review. The first author (AKJT) performed the database searches, screened articles based on the inclusion and exclusion criteria, and extracted information. The processes were verified by FM.

##

## **Supplementary Figure 1: Flow diagram of literature search addressing key practices and challenges in addressing TB among older adults in the Western Pacific Region**


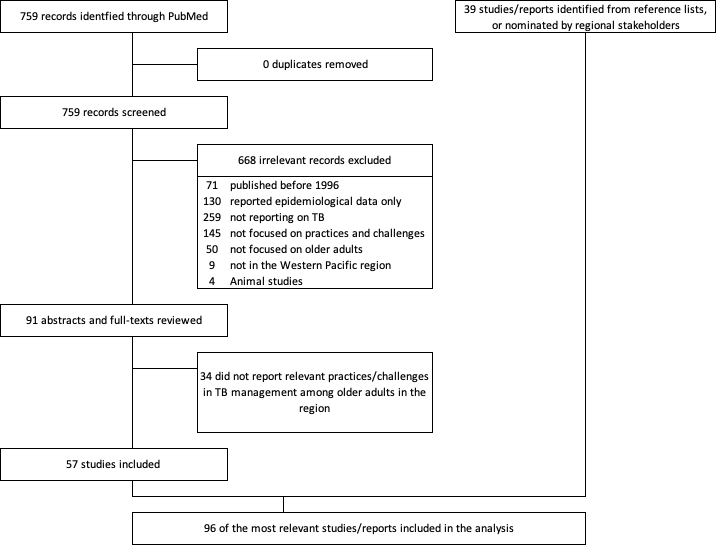


**References**

1 World Health Organization Regional Office for the Western Pacific. World Health Organization: Western Pacific - where we work. 2009. https://www.who.int/westernpacific/about/where-we-work (accessed April 18, 2022).
